# Do Green Finance Policies Foster Environmental, Social, and Governance Performance of Corporate?

**DOI:** 10.3390/ijerph192214920

**Published:** 2022-11-13

**Authors:** Xingshuai Wang, Ehsan Elahi, Zainab Khalid

**Affiliations:** 1 School of Economics, Shandong University of Technology, Zibo 255000, China; 2 School of Economics and Management, Southeast University, Nanjing 210096, China

**Keywords:** carbon neutrality, green finance, green credit, corporate ESG, environmental regulation

## Abstract

The green finance policy is crucial for enterprises to participate in environmental governance actively. Taking the “Green Credit Guidelines” issued by China in 2012 as a quasi-natural experiment, this study investigated the impact of green finance policies on corporate environmental, social, and governance (ESG) performance by using a continuous Difference-in-Differences (DID) model based on the data of listed companies from 2006 to 2020. The conclusions are: (1) The green finance policy significantly improves corporate ESG, but the effects vary across enterprises. (2) The policy has encouraged enterprises to develop and adopt green products and technologies. Still, it has not had a positive effect on the treatment of enterprise pollutant emissions because the implementation of the policy makes enterprises pay more attention to front-end risk control than pollution treatment after production. (3) Research results have heterogeneity. The impact of green finance policies on enterprises at different levels of environmental regulation is different. Enterprises in areas with high intensity of environmental regulation are more vulnerable to green credit. The conclusion of this paper helps improve the green finance policy system, enhance the awareness and level of corporate ESG, and strengthen the collaborative governance of policies and enterprises on environmental issues in combination with the mandatory environmental regulations and incentive mechanisms to promote the green development of enterprises and realize the goal of carbon neutrality.

## 1. Introduction

**Figure 1:**
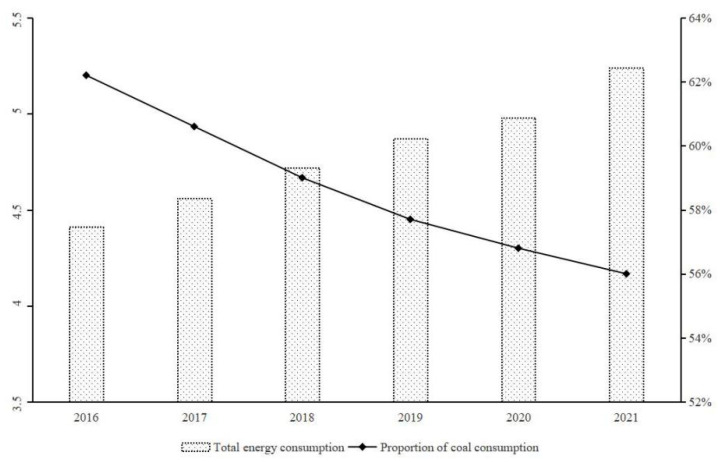
The trend of China’s energy consumption.

Scale-driven economic growth has led to excessive energy consumption and pollution emissions [1,2]. Problems, such as over-exploitation of resources, environmental pollution, and abnormal climate, have become huge obstacles to sustainable economic development [3,4]. According to the statistics released by the National Bureau of Statistics of China, in 2021, China’s total energy consumption was 5.24 billion tons of standard coal, an increase of 5.2% over the previous year (Figure 1). Coal consumption accounted for 56.0% of the total energy consumption. China is still the world’s largest coal consumer and greenhouse gas emitter. According to calculations based on China Emission Accounts and Datasets (CEADs, https://www.ceads.net.cn (accessed on 10 September 2022)), in 2021, China’s total carbon emissions exceeded 10.3 billion tons, accounting for about 27% of the total global carbon emissions, close to the sum of the United States, the European Union, and Japan. The task of achieving carbon neutrality by 2060 is extremely arduous. In recent years, China has rapidly developed green finance with carbon reduction, environmental protection, and sustainable development. According to the statistical data of the People’s Bank of China, by the end of 2021, the balance of China’s green loans was CNY 15.9 trillion, an increase of 33%, ranking first in the world. As an important policy tool of modern environmental governance, green finance not only has the characteristics of market-oriented environmental regulation but also has the function of resource allocation of the financial sector. Promoting the development of green finance can reduce the credit rationing of ‘two high industries’ (high energy-consuming and high-emissions), improve the industrial structure, and accelerate the transformation of production mode to cleaner production [5,6]. The goal of environmental governance is to change the behavior choice of enterprises. Only when more enterprises take the initiative and are willing to undertake ESG can carbon neutrality be finally achieved.

As the primary green financial tool, green credit has gradually attracted the attention of Chinese government departments. In 2012, the China Banking Regulatory Commission issued the “notice on printing and distributing green credit guidelines”, which put forward more explicit and specific requirements for the banking industry, from green credit organization management, process management, internal control, and information disclosure. It emphasized that financial institutions should take energy conservation, emission reduction, environmental protection, and other factors as important bases for credit decisions, not grant credit to customers that may bring risks to the environment and society, and increase credit support for pollution control and resource conservation projects. This policy provides an excellent opportunity to identify the policy effect of green finance. The main reason is that data availability makes it difficult to accurately measure green bonds and stocks. With green credit gradually becoming China’s most important green financial tool, it is reasonable to use it as a proxy variable of green finance.

Under the traditional financial framework, there is a contradiction between the corporate ESG and the profit maximization goal [7]. Enterprises that do not undertake or rarely undertake ESG may achieve better market performance [8,9]. Due to the lack of assessment of the enterprise’s environmental level and supervision of the investment flow, the advantages of green enterprises in the traditional credit market are not obvious. The green finance policy requires enterprises to provide environmental information, which can alleviate the adverse selection and moral hazard caused by information asymmetry [10]. When there are no external constraints, enterprises mainly consider using limited resources to maximize profits and generally not be motivated to fulfill ESG. The implementation of the green finance policy enables the financial sector to incorporate environmental factors into enterprise credit granting and project management and grant more credit resources to green projects. However, due to the asymmetry of information in the capital market, it is difficult to observe the actual green level of enterprises. Therefore, enterprises must send signals to financial institutions through their green behaviors to ensure that they have an advantage in credit activities. Among them, the most convenient way is to undertake ESG or carry out environmental information disclosure [11,12] and send “green signals” to financial institutions in exchange for favorable conditions in the credit market, thus green finance can more effectively encourage financial institutions and enterprises to assume more social responsibilities. However, some studies believe that the effectiveness of green finance depends on environmental regulation and the response of enterprises to policies, and the willingness of enterprises to assume social responsibility directly impacts the implementation effect of policies [13]. Therefore, it is important evidence to evaluate the micro effect of green finance to explore whether green finance can have a practical impact on corporate behavior, analyze the regulatory effect of corporate behavior on green finance policy, and clarify the specific ways of green finance affecting corporate ESG behavior [14].

## 2. Literature Review

### 2.1. Environmental Regulation

Environmental regulation is formulated to solve the problem of environmental pollution and is a policy tool to regulate enterprises’ production and business activities [15]. The researchers used the ‘pollution information transparency index (PITI)’ to measure the government’s environmental regulation and found that the improvement of the effectiveness of government environmental regulation will increase the contribution of enterprise transformation and upgrading to environmental governance [16]. From the perspective of green governance of environmental regulation on micro-enterprises, existing studies mainly explore the relationship between environmental regulation and green innovation of enterprises. For example, researchers took the pilot emission trading policy as an example to empirically study the relationship between the implementation of the policy and green innovation and found that the pilot emission trading policy induced green innovation of enterprises, and the inducing effect was mainly on green invention patents [17]. Sewage charges have a “reverse force” effect on the green innovation ability of enterprises, while environmental protection subsidies have a “crowding out” effect on the green innovation of enterprises [18]. Environmental regulation can improve the total factor productivity of enterprises in the long run [19]. The credibility of the mechanism’s implementation scheme and the policy’s accuracy would affect the realization of low-emission technologies [20]. Under environmental regulation, enterprises will increase their environmental protection investment [21]. Many scholars have proved in their own research that environmental regulation is conducive to promoting the innovation level of enterprises [22,23]. Environmental regulation can improve all enterprises’ green factor energy efficiency by increasing green innovation [24,25].

The research on environmental regulation focuses more on the relationship between environmental regulation and macro-level factors, such as total factor productivity, efficiency, industrial competitiveness, and industrial structure at the regional and industrial levels. Some studies focus on the relationship between environmental regulation and enterprise innovation or green technology innovation but less on environmental regulation and corporate ESG.

### 2.2. Green Finance

Green finance refers to economic activities that support environmental improvement, climate change, and the economical and efficient use of resources, including financial services provided for investment, project operation, and risk management in areas such as environmental protection, energy conservation, clean energy, green transportation, and green buildings. Green finance has a broader impact on the environment, society, and enterprises [26]. The government’s strong environmental protection policies must guarantee the effective implementation of green finance [27]. Green finance can help improve the efficiency of capital allocation, guide capital to enter the green and environmental protection industries, and thus play a role in adjusting industrial structure and promoting industrial upgrading [28]. In addition, it can reduce environmental pollution and promote sustainable economic development and carbon neutrality [29,30,31]. Green finance is an essential tool for environmental protection and its development can achieve sustainable economic development while maintaining a low level of pollution emissions [32].

The green finance policy helps to limit the credit scale of heavily polluting industries, promote green innovation, improve the environment, and radiate to surrounding areas. The empirical results show that green finance can significantly reduce the emissions of air, water, and solid pollutants, affirming the role of green credit policies in improving environmental quality [33]. However, green finance reflects the heterogeneity in different market environments and legal levels and heterogeneous effects among enterprises with different levels of environmental performance. This policy reduces the financing cost of environmentally friendly enterprises and increases the financing burden of high-pollution enterprises. In general, scholars have reached a consensus on the positive role of green finance in energy conservation and emission reduction, pollution control, and carbon neutrality.

The green credit policy has the dual attributes of environmental regulation and financial resource allocation. The possibility of enterprises participating in environmental and social responsibility will be effectively improved due to the behavioral motivation of obtaining credit funds. In previous studies, the main channel for green credit policy to affect enterprise behavior is the increase of capital cost caused by financing constraints, which is reflected in the additional interest enterprises pay to obtain credit. It also leads to the “financing channel effect”, where financial institutions set certain environmental thresholds, making heavily polluting enterprises unable to get credit support [34]. Enterprises need to submit environmental and social governance risk reports before obtaining loans and regularly disclose environmental and social governance disclosures after obtaining loans. Green credit policy can exclude polluting enterprises from the scope of credit support, thus forcing enterprises to carry out green transformation and forcing management to pay attention to corporate environmental and social responsibility governance.

### 2.3. Corporate ESG

External pressure is an important factor that urges enterprises to perform ESG. Government regulatory pressure is an important driving force for enterprises to undertake environmental responsibility. The stricter government regulation, the higher the environmental responsibility consciousness of enterprises [35]. The government’s attention to environmental regulation is conducive to promoting enterprises to fulfill their ESG [36], and enterprises will fulfill their ESG under the pressure of public policies [37]. Moreover, the performance of ESG can transmit a signal to the outside that is conducive to enterprise financing [38]. Performing ESG can reduce the enterprise’s risk and financing cost [39] and help enterprises out of financial difficulties [40]. When managers make and disclose a green investment and emphasize its social benefits, investors will also have positive reactions [41]. Listed companies are more inclined to assume ESG and disclose relevant information to transmit more positive signals to the capital market, to strengthen exchanges with investors, and further improve the company’s value [42]. The greater the pressure of environmental regulation, the more likely enterprises are to assume ESG by internalizing environmental costs [43]. Combining green finance, ESG theory, and green growth theory, enterprises’ performance of ESG is also conducive to improving the quality of economic growth [44]. Much empirical evidence shows that enterprises regard ESG as an investment opportunity in more cases [45].

The existing literature has laid a good foundation for studying the micro effects of green finance policies. Still, only a few studies have focused on the impact of green finance on corporate ESG behavior [12,46]. The measurement of ESG is relatively simple, which cannot reflect the behavior choice and green governance of enterprises under the green finance policy [47]. To sum up, the contributions of this paper are mainly reflected in enriching the research on the micro effects of green finance policies and increasing the attention to corporate ESG. This paper analyzes the internal factors of green finance affecting enterprises and studies the impact of green finance policies on corporate ESG in more detail.

## 3. Materials and Research Design

### 3.1. Materials

This paper takes China’s A-share listed companies from 2006 to 2020 as the sample. The data sources mainly include the following parts: data of listed companies, the basic information, financial statements, and other data of all listed companies are obtained from the Chinese Research Data Services Platform (CNRDS), and relevant indicators such as control variables and financing costs are calculated and eliminate the data with missing severe financial indicators. Data of industrial pollution emission is obtained from China Environmental Statistics Yearbook over the years. After matching the above data, the data of 1196 listed companies were finally obtained.

### 3.2. Modeling and Variable Definition

Under the modern environmental governance system, the behavior choice of enterprises can directly change the industrial structure and production mode and ultimately determine the implementation effect of environmental regulation. Green credit can encourage enterprises to reduce pollution emissions and improve environmental and social governance. For example, in 2014, China formulated “The Key Assessment Indicators for the Implementation of Green Credit,” which divided the production and operation activities and industry scope of enterprises that pollute the environment, providing a basis for the banking industry to carry out the customer selection and risk management of green credit. The policy impact of green credit runs through the whole process of enterprise production. In the financing stage, the credit quota is inclined to green enterprises. In the production process, financial institutions can carry out post-loan management according to the green credit policy to promote the green production of enterprises. After the production is completed, the green audit can be carried out further. Therefore, compared with traditional environmental regulation, green credit policy has a more significant structural effect and a more complete environmental and social governance cycle.

This paper uses the ‘notice on printing and distributing green credit guidelines’ issued in 2012 as a quasi-natural experiment. A continuous difference-in-differences (DID) method has been used to investigate the impact of green finance, especially green credit policies, on corporate ESG [48]. The definition of treatment group and control group in existing research mainly divides enterprises into high-pollution enterprises and low-pollution enterprises. It compares the differences between groups, which is insufficient to identify treatment groups. This study uses the restrictive industries of green credit policy to define the setting method of experimental groups.

On the one hand, it excludes the non-restrictive industries from being defined as experimental groups. On the other hand, it also depicts the size of the experimental groups affected by the policy. The basic idea of the DID model is to identify the average processing effect of the policy by using the difference in the intensity of the impact of the policy between the experimental group and the control group. The model is as follows:

(1) $${\mathrm{ESG}}_{jft}=\alpha_{0}+\alpha_{1}{\mathrm{Green}}_{jt}+\alpha_{2}{\mathrm{Industry}}_{jt}+\alpha_{3}{\mathrm{Control}}_{ft}+\gamma_{f}+\gamma_{j}+\gamma_{t}+\varepsilon_{jft}$$

where f means enterprise, j means industry, and t means year. The dependent variable ${\mathrm{ESG}}_{jft}$ represents the corporate ESG behavior, and ${\mathrm{Green}}_{jt}$ is the main independent variables to measure the extent to which the experimental group is affected by the green finance policy. ${\mathrm{Industry}}_{jt}$ is a virtual variable that distinguishes whether the green finance policy restricts the industry, and ${\mathrm{Control}}_{ft}$ represents other control variables. In addition, the fixed effect of individual enterprise, industry effect, and time effect are added to the model, and $\varepsilon_{jft}$ represents the interference term which is assumed to be normally distributed at zero mean value [49,50,51] nd constant variance [52,53,54].

#### 3.2.1. The Dependent Variable

This paper mainly investigates the impact of green finance, represented by green credit policy, on two kinds of corporate ESG behaviors. Green technology (GT) is measured by the indicator ‘whether the enterprise develops or applies innovative products or technologies beneficial to the environment. If this variable takes 1, it indicates that the enterprise has allocated corresponding funds for purchasing environmentally beneficial technologies and product R&D. Pollution emission (PE) is measured by the indicator “whether the enterprise has taken measures to reduce the emission of industrial solid waste, industrial waste gas, industrial smoke, and dust discharge and industrial wastewater discharge”, which reflects the treatment of pollutant emissions by the enterprise.

#### 3.2.2. Main Independent Variable

Industry is a sub item of traditional DID. If the enterprise is a green credit-restricted industry and the sample year is 2012 or later, take 1. ${\mathrm{Green}}_{\mathrm{jt}}$ is the cross-term of ${\mathrm{Industry}}_{\mathrm{jt}}$ and the extent to which the green credit policy affects the enterprise. Its estimated coefficient is the policy effect. $\alpha_{1}$ > 0 indicates that green credit policies promote the improvement of corporate environmental and social governance, $\alpha_{1}$ < 0 means that policies inhibit the improvement of corporate environmental and social governance, and $\alpha_{1}$ = 0 indicates that the policy effect is not obvious. It mainly measures the degree of impact by the green credit policy. It uses the weighted average of pollutant emission reduction levels of various industries to measure the intensity of the industry affected by the policy. This paper selects four kinds of pollutants: industrial solid waste, industrial waste gas, industrial smoke, and dust discharge and industrial wastewater discharge, and the overall change of pollutant discharge in each industry is determined by standardized methods. The main sources of industrial pollutant data are the ‘China Environmental Statistics Yearbook’ and ‘China Statistical Yearbook’ over the years. The industries restricted by green credit policies match those of listed companies.

#### 3.2.3. The Control Variable

This paper introduces a series of control variables to control other characteristics that affect corporate ESG. The size of the enterprise (Size) is measured by the natural logarithm of total annual assets. The asset liability ratio (Lev) is the total liabilities divided by total assets at the end of the year. ROA is the net profit divided by the average balance of total assets. The cash flow ratio (Cashflow) is the cash flow generated from operating activities divided by total assets. The increase rate of primary business revenue (Growth) is the ratio of this year’s operating revenue to the previous year’s operating revenue minus 1. The enterprise nature (SOE) is the ownership nature of the enterprise; the state-owned enterprise takes 1.

### 3.3. Descriptive Statistics

Table 1 shows the descriptive statistical results for the above variables. To avoid the heteroscedastic problem and narrow the intra-group and inter-group differences of variables, the variable of enterprise size is processed by logarithmic transformation.

## 4. Empirical Analysis

### 4.1. Parallel Trend and Policy Time Uniqueness

The premise that the results of the DID model are effective is that the parallel trend hypothesis of the experimental group and the control group is established and the policy time is determined. Therefore, before the benchmark regression, the event research method is used to test the difference between the experimental group and the control group before the start of the policy, and the uniqueness of the policy time is tested to ensure that there is no policy effect before 2012 (Table 2).

**Figure 2:**
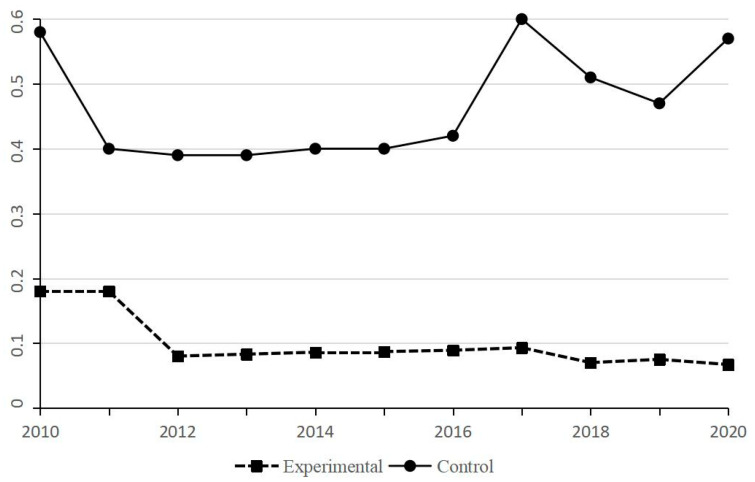
Parallel trend test results.

To ensure that the parallel trend hypothesis and the uniqueness of the policy time point are established, this paper conducts two tests: First, test whether the parallel trend hypothesis is established. As shown in Figure 2, by comparing the two data groups, it is confirmed that the experimental and control groups’ development trends before the policy are basically the same, with the same development trend, indicating that the model meets the common trend assumption. Second, only the samples before the start of the policy are retained, and the start time of the policy is assumed to be 3 years (2009) and 4 years (2008), respectively. Since the start time of the policy is “false”, it is expected that the green credit policy will not significantly impact all enterprises’ environmental and social responsibility behaviors. The results are basically in line with expectations; before the policy started, the green credit policy had no significant impact on enterprises’ two corporate ESG behaviors. It is reasonable to take 2012 as the policy time point.

### 4.2. Benchmark Regression

Table 3 reports the benchmark regression results of Formula (1). columns (1)–(2) show the results without adding industry and time-fixed effects, and columns (3)–(4) show the results with adding all control variables and fixed effects. The results show that the green credit policy has a different impact on two different corporate ESG. Among them, the green credit policy encourages enterprises to develop or use green products or technologies beneficial to the environment. With the increase of policy intensity, the probability increases correspondingly and has significant economic significance. However, the restricted industries of green credit are not significant, which indicates that there are certain differences in environmental performance among industries, and the intensity of their influence by policies is not the same.

Consistent with the research conclusions of [48,55], the estimation results in Table 3 show that green credit does not have a positive effect on the treatment of enterprise pollutant emissions because green credit management mainly focuses on risk control before the start of the project, rather than pollution treatment after the end of production. In recent years, with the continuous improvement of the level of corporate ESG, the green credit policy has promoted the transformation of the corporate ESG structure. Enterprises gradually use and develop green products or technologies that are beneficial to the environment to replace the pollution discharge treatment with the high cost and low efficiency, and ultimately reduce the possibility of enterprises carrying out pollution treatment. In general, the green credit policy has improved the corporate ESG, but the effects of different governance methods are different.

### 4.3. Robustness Test

#### 4.3.1. PSM/EBM-DID Estimation

Propensity score matching (PSM) and entropy balance matching (EBM) are used to correct the difference between samples. Refer to the matching method of [25], and control variables were used as covariates for matching. columns (1)–(2) of Table 4 are the matching estimation results of the propensity score, and columns (3)–(4) are the matching estimation results of entropy balance. The DID estimation results after matching show that the estimation results are significant at 1%. The impact of green finance on the use of green products and technologies by enterprises is still positive. At the same time, the treatment of pollutant emissions is adverse, consistent with the results of benchmark regression.

#### 4.3.2. Placebo Test

This paper takes non-ESG behaviors as independent variables for analysis. column (1) of Table 5 measures whether the enterprise has projects supporting charitable donations, column (2) measures whether the company has policies or standards to promote employment, column (3) measures whether the company and business partners have established strategic sharing mechanisms and platforms, and column (4) estimates whether the enterprise has the concept and system guarantee of honest operation and fair competition. It is expected that these ESG behaviors will not be affected by the green credit policy. The results show that from the significance perspective, which is not significant at a 10% significance level. The green credit policy has no impact on corporate ESG behavior unrelated to the environment.

#### 4.3.3. Sample Selection

The Heckman selection model [56] is used to test whether sample selectivity bias is caused by the non-random behavior of enterprises. By calculating ‘Inverse Mill’s Ratio (IMR)’, IMR is added to the model to eliminate sample selectivity bias. Compared with the benchmark regression results, the results after eliminating the sample selection errors are still significant at 5% significance level (Table 6). Therefore, the sample selection problem does not lead to obvious estimation bias.

#### 4.3.4. Exclude the Impact of Other Policies

This paper excludes other policy factors from two aspects: Examine the impact of the ‘Environmental Protection Law’ (EPL) on corporate ESG. In 2015, China formally implemented the EPL, which stipulates the responsibility of enterprises to prevent and control environmental pollution, which may have a significant impact on corporate ESG. In columns (1) and (2) of Table 7, this paper excludes the impact of the EPL by introducing dummy variables of years after 2015. Moreover, it examines the role of other green finance policies. Since 2016, China has set up ‘green finance reform and innovation pilot areas’ in six provinces. The development of green finance in the pilot areas may differ from that in other regions. In columns (3) and (4) of Table 7, the impact of the policy is eliminated by deleting the sample of provinces that have established green finance reform and innovation pilot zones. After testing, it is consistent with the benchmark regression results.

### 4.4. Heterogeneity Test

Green finance, as a market-oriented environmental regulation, pays more attention to the economic incentives of corporate ESG than the “punishment effect” of policies. Although some empirical studies have shown that there are incentives for enterprises to perform ESG, some studies believe that without strict environmental law enforcement, it is difficult to implement policies effectively [57]. If the pollution behavior is not paid enough attention or punished lightly, the motivation of enterprises to perform environmental and social governance will also be reduced. Therefore, the effects of policies are also different for enterprises in other regions with different environmental regulation strengths. It is necessary to discuss the impact of green finance in the different areas with different environmental regulation strengths from the heterogeneity perspective.

Table 8 reports the estimated results under different levels of environmental regulation; columns (1)–(2) are the estimation results of low environmental regulation areas, and columns (3)–(4) are the estimation results of high environmental regulation areas. The results show that in areas with low intensity of environmental regulation, the impact of green finance on the development of green products and the use of green technologies of enterprises is not obvious. The treatment of pollutant emissions by enterprises has also decreased significantly, which indicates that when the level of environmental regulation is low, enterprises are only inclined to carry out low-cost ESG. However, when environmental regulation intensifies, green finance has a significant incentive on enterprises to develop green products and use green technologies. Still, it has no significant impact on treating pollutant emissions. This shows that in areas with high intensity of environmental regulation, enterprises will be more inclined to carry out more effective green product development and use of green technologies to obtain green financial policy preferences. Instead of using the traditional means of pollutant emission control, this shows the strategic choice of enterprises in the face of green financial policies. Based on the above explanation, it shows that strict environmental regulation is the prerequisite for the effective implementation of green financial policies.

## 5. Conclusions and Policy Recommendations

This paper uses the data of China’s listed companies to evaluate the impact of green finance on corporate ESG. The main conclusions are as follows: green finance policies have generally improved corporate ESG, but the effects of different types of governance have specific differences. Green finance has encouraged enterprises to develop or use green products or technologies that benefit the environment. Still, it has not positively affected the treatment of enterprise pollutant emissions because implementing green finance policies has made enterprises pay more attention to front-end risk control than pollution treatment after production. Moreover, the influence of green finance policy under different levels of environmental regulation is different. When the intensity of environmental regulation is high, green finance has a significant incentive effect on enterprises to develop green products and use green technologies but has no significant impact on the treatment of pollutant emissions. This shows that in areas with high intensity of environmental regulation, enterprises will be more inclined to carry out more effective green product development and use of green technologies to obtain green financial policy preferences instead of using traditional means of pollutant discharge control. The research conclusions of this paper mainly include the following policy recommendations:

1. Further improve the green finance policy system. The government and financial institutions should improve the green finance recognition standards, evaluation systems, regulatory mechanisms, and environmental information disclosure systems to help enterprises comprehensively enhance the level of ESG. Establish an evaluation mechanism for implementing green finance transactions and conduct a qualitative and quantitative evaluation on the implementation effect of green finance business implemented by financial institutions.
2. Enhance the awareness of corporate ESG and strengthen government and enterprises’ coordinated governance of environmental issues. An important prerequisite for the green finance policy to achieve its effect is the behavior choice of enterprises. The attention of enterprises may lead to different policy performances. The higher the attention of enterprises to ESG, the more likely they are to comply with the policy requirements of green finance. Therefore, it is necessary to improve the awareness of corporate ESG through green financial policies. On the one hand, we should change the cost of pollution activities through policy guidance to encourage enterprises to pay more attention to environmental problems in production. On the other hand, let enterprises intuitively understand the implementation standards and specific forms of green finance policies and participate in environmental governance together to understand the policy objectives.
3. Reasonably match mandatory environmental regulation and green financial policies and establish an incentive mechanism for ESG. Environmental regulation without incentive mechanisms is also difficult to achieve green and coordinated development of the industry, and the effectiveness of green financial policy needs the support of other conditions. In the future, improve the strictness of mandatory environmental regulation, strengthen the link between environmental protection and official performance, and avoid local governments’ inaction in environmental regulation. Moreover, establish an incentive mechanism for green financial policies, implement differentiated incentives, and give more support and preferential treatment to enterprises with better ESG, which is conducive to improving the implementation effect of green financial policies.

## Figures and Tables

**Table 1:** Descriptive statistics.

Variables	N	Mean	S.D.	Min.	Max.
GT	7486	0.47	0.499	0	1
PE	7486	0.18	0.384	0	1
Green	7486	−0.049	0.431	−3.846	0.614
Industry	7486	0.253	0.435	0	1
Size	7486	23.09	1.404	19.35	26.4
Lev	7486	0.442	0.375	0.027	0.99
ROA	7486	0.047	0.059	−0.415	0.245
Cashflow	7486	0.053	0.070	−0.224	0.283
Growth	7486	0.251	0.776	−0.732	4.806
SOE	7486	0.568	0.495	0	1

**Table 2:** Policy time uniqueness.

	GT	PE	GT	PE
	(1)	(2)	(3)	(4)
Green_placebo2009	0.024	0.009		
	(−0.036)	(−0.009)		
Green_placebo2008			0.115	0.003
			(−0.207)	(−0.005)
cons	−0.325	−0.015	−0.232	−0.014
	(−1.766)	(−0.072)	(−1.814)	(−0.078)
N	1500	1500	1500	1500
R^2^	0.474	0.971	0.474	0.971

Standard errors are given in the parentheses.

**Table 3:** Benchmark regression.

	GT	PE	GT	PE
	(1)	(2)	(3)	(4)
${\mathrm{Green}}_{jt}$	0.066 ***	−0.035 **	0.063 ***	−0.037 **
	(−0.020)	(−0.015)	(−0.020)	(−0.015)
Industry	0.010	0.048	−0.032	0.049
	(−0.019)	(−0.034)	(−0.038)	(−0.033)
Cons	−1.405 ***	−0.163	−0.708	0.361
	(−0.378)	(−0.327)	(−0.471)	(−0.277)
Enterprises FE	Yes	Yes	Yes	Yes
Industry FE	No	No	Yes	Yes
Year FE	No	No	Yes	Yes
N	7300	7300	7300	7300
R^2^	0.399	0.854	0.435	0.857

Standard errors are given in the parentheses. ** *p* < 0.05, *** *p* < 0.01.

**Table 4:** PSM/EBM-DID estimation results.

	GT	PE	GT	PE
	(1)	(2)	(3)	(4)
${\mathrm{Green}}_{jt}$	0.067 ***	−0.039 **	0.063 ***	−0.040 ***
	(−0.016)	(−0.013)	(−0.019)	(−0.013)
Industry	−0.072	0.043	−0.034	0.050
	(−0.043)	(−0.033)	(−0.037)	(−0.032)
Size	0.045 **	−0.007	0.036 ***	−0.005
	(−0.019)	(−0.013)	(−0.012)	(−0.016)
Lev	0.032	−0.001	0.032	−0.006
	(−0.026)	(−0.008)	(−0.020)	(−0.008)
ROA	−0.219	0.093	−0.241 *	0.129
	(−0.163)	(−0.106)	(−0.123)	(−0.089)
Cashflow	−0.090	0.001	0.006	0.046
	(−0.092)	(−0.047)	(−0.173)	(−0.055)
Growth	0.080 ***	0.007	0.105 ***	0.007
	(−0.012)	(−0.005)	(−0.006)	(−0.007)
SOE	−0.096 *	0.025	−0.066	0.042
	(−0.049)	(−0.038)	(−0.057)	(−0.038)
Cons	−0.505	0.317	−0.333	0.315
	(−0.459)	(−0.305)	(−0.310)	(−0.360)
Enterprises FE	Yes	Yes	Yes	Yes
Industry FE	Yes	Yes	Yes	Yes
Year FE	Yes	Yes	Yes	Yes
N	6000	6000	7300	7300
R^2^	0.491	0.875	0.423	0.860

Standard errors are given in the parentheses. * *p* < 0.1, ** *p* < 0.05, *** *p* < 0.01.

**Table 5:** Placebo test.

	Charitable Donations	Promote Employment	Strategic Sharing	Honest Operation
	(1)	(2)	(3)	(4)
${\mathrm{Green}}_{jt}$	0.004	−0.004	0.064	0.016
	(−0.005)	(−0.019)	(−0.058)	(−0.027)
Industry	0.015	0.035 *	−0.031	−0.013
	(−0.020)	(−0.019)	(−0.042)	(−0.019)
Size	0.045 **	0.066 ***	0.005	0.020 *
	(−0.017)	(−0.013)	(−0.027)	(−0.011)
Lev	0.022	0.018	0.046 *	0.005
	(−0.019)	(−0.014)	(−0.022)	(−0.015)
ROA	0.234	0.282 **	0.077	−0.073
	(−0.155)	(−0.123)	(−0.194)	(−0.114)
Cashflow	0.088	−0.129 **	−0.095	0.014
	(−0.073)	(−0.058)	(−0.114)	(−0.069)
Growth	0.000	0.007	0.008	−0.002
	(−0.003)	(−0.007)	(−0.01)	(−0.006)
SOE	−0.016	0.034	0.113	−0.021
	(−0.035)	(−0.048)	(−0.069)	(−0.029)
Cons	−0.702 *	−1.180 ***	0.256	0.401
	(−0.392)	(−0.279)	(−0.639)	(−0.258)
Enterprises FE	Yes	Yes	Yes	Yes
Industry FE	Yes	Yes	Yes	Yes
Year FE	Yes	Yes	Yes	Yes
N	7300	7300	6500	6500
R^2^	0.549	0.456	0.417	0.353

Standard errors are given in the parentheses. * *p* < 0.1, ** *p* < 0.05, *** *p* < 0.01.

**Table 6:** Heckman selection model.

	GT	PE
	(1)	(2)
${\mathrm{Green}}_{jt}$	0.058 ***	−0.031 **
	(−0.017)	(−0.012)
Industry	−0.025	0.047
	(−0.028)	(−0.033)
IMR1	0.194 ***	
	(−0.039)	
IMR2		−0.014
		(−0.021)
Cons	−5.398 ***	0.684 *
	(−0.949)	(−0.368)
Enterprises FE	Yes	Yes
Industry FE	Yes	Yes
Year FE	Yes	Yes
N	7300	7300
R^2^	0.441	0.859

Standard errors are given in the parentheses. * *p* < 0.1, ** *p* < 0.05, *** *p* < 0.01.

**Table 7:** Exclude the impact of other policies.

	GT	PE	GT	PE
	(1)	(2)	(3)	(4)
${\mathrm{Green}}_{jt}$	0.065 ***	−0.036 **	0.080 **	−0.036 *
	(−0.019)	(−0.014)	(−0.031)	(−0.017)
Industry	−0.019	0.038	−0.028	0.054
	(−0.031)	(−0.030)	(−0.043)	(−0.037)
EPL	−0.010	0.032 **		
	(−0.024)	(−0.012)		
Cons	−20.337	−2.795	−0.525	0.390
	(−13.716)	(−4.769)	(−0.493)	(−0.367)
N	7300	7300	5400	5400
R^2^	0.400	0.855	0.433	0.846

Standard errors are given in the parentheses. * *p* < 0.1, ** *p* < 0.05, *** *p* < 0.01.

**Table 8:** Heterogeneity analysis results.

	Low Environmental Regulation Areas		High Environmental Regulation Areas	
	GT	PE	GT	PE
	(1)	(2)	(3)	(4)
${\mathrm{Green}}_{jt}$	0.096	−0.042 **	0.042 ***	−0.034
	(−0.068)	(−0.018)	(−0.008)	(−0.020)
Industry	−0.122	0.047	0.018	0.054
	(−0.076)	(−0.035)	(−0.044)	(−0.032)
Cons	−0.570	0.395	−0.619	0.330 *
	(−0.931)	(−0.540)	(−0.367)	(−0.165)
Enterprises FE	Yes	Yes	Yes	Yes
Industry FE	Yes	Yes	Yes	Yes
Year FE	Yes	Yes	Yes	Yes
N	2900	2900	4500	4500
R^2^	0.446	0.873	0.438	0.846

Standard errors are given in the parentheses. * *p* < 0.1, ** *p* < 0.05, *** *p* < 0.01.

## Data Availability

The data used to support the findings of this study are available from the corresponding author upon request.
